# Relevance of Caspase-1 and Nlrp3 Inflammasome on Inflammatory Bone Resorption in A Murine Model of Periodontitis

**DOI:** 10.1038/s41598-020-64685-y

**Published:** 2020-05-08

**Authors:** Fernanda R G. Rocha, Andrea E. Delitto, Joao A. Chaves de Souza, Laura A. González-Maldonado, Shannon M. Wallet, Carlos Rossa Junior

**Affiliations:** 1 Department of Oral Biology, College of Dentistry, University of Florida, Gainesville, FL USA; 2 Department of Diagnosis and Surgery, UNESP-State University of Sao Paulo, School of Dentistry at Araraquara, Araraquara, SP Brazil; 3 Department of Physical Therapy, University of Florida Health Science Center, Gainesville, FL USA; 4 Department of Stomatology, School of Dentistry, Federal University of Goias (UFG), Goiania, GO Brazil; 5 Department of Oral and Craniofacial Health Sciences, School of Dentistry, University of North Carolina, Chapel Hill, NC USA

**Keywords:** Inflammatory diseases, Inflammasome

## Abstract

This study investigates the role of NLRP3 inflammasome and its main effector Caspase-1 in inflammation and alveolar bone resorption associated with periodontitis. Heat-killed *Aggregatibacter actinomycetemcomitans* (Aa) was injected 3x/week (4 weeks) into gingival tissues of wild-type (WT), *Nlrp3*-KO and *Caspase1*-KO mice. Bone resorption was measured by µCT and osteoclast number was determined by tartrate-resistant acid phosphatase (TRAP) staining. Inflammation was assessed histologically (H/E staining and immunofluorescence of CD45 and Ly6G). *In vitro* studies determined the influence of Nlrp3 and Caspase-1 in Rankl-induced osteoclast differentiation and activity and on LPS-induced expression of inflammation-associated genes. Bone resorption was significantly reduced in *Casp1*-KO but not in *Nlrp3*-KO mice. *Casp1*-KO mice had increased in osteoclast numbers, whereas the inflammatory infiltrate or on gene expression were similar to those of WT and *Nlrp3*-KO mice. Strikingly, osteoclasts differentiated from *Nlrp3*-deficient macrophages had increased resorbing activity *in vitro*. LPS-induced expression of Il-10, Il-12 and Tnf-α was significantly reduced in *Nlrp3*- and *Casp1*-deficient macrophages. As an inceptive study, these results suggest that Nlrp3 inflammasome does not play a significant role in inflammation and bone resorption *in vivo* and that Caspase-1 has a pro-resorptive role in experimental periodontal disease.

## Introduction

Inflammasomes are cytosolic multiprotein complexes activated in response to various stimuli, including both microbial-associated molecular patterns (MAMPs) and damage-associated molecular patterns (DAMPs). A major biological function is the final processing of various inflammation-associated cytokines, including IL-1β, IL-18 and IL-33, into the biologically active form^1^. The relevance of inflammasomes to the immune response is demonstrated by the association between mutations in the genes encoding their protein components and autoimmune inflammatory conditions, or dysregulation of the immune response^2,3^. NLRP3 is the most studied inflammasome and has been associated with various diseases and conditions characterized by chronic inflammation, including gout, cancer, type 2 diabetes and rheumatoid arthritis, besides periodontal diseases^1,4–6^.

Periodontal disease is a chronic inflammatory condition induced by microbial insult derived from a highly complex dental biofilm and is the most prevalent lytic lesion of bone in humans^7,8^. This condition represents and excellent model to study the role of inflammasomes due to the abundance of MAMPs and DAMPs and the elevated proportion of macrophages in the tissue microenvironment.

Interestingly, there is a relative scarcity of information on the biological roles of inflammasomes derived from clinical or pre-clinical studies in periodontal disease models. Increased expression of IL-1β and IL-18 in the gingival tissues and gingival crevicular fluid of patients with various forms of periodontal disease^9–11^ correlates positively with increased expression of NLRP3 mRNA in this microenvironment^12^, suggesting the participation of this inflammasome in the pathogenesis of periodontal disease. The possible involvement of NLRP3 inflammasome is further suggested by the increased expression of NLRP3 in the oral epithelium^13^ and in the saliva of periodontal disease patients^14^.

The canonical effector protein activated downstream of most inflammasomes is the protease Caspase-1, which cleaves the pro-form of inflammatory cytokines IL-1β, IL-18 and IL-33, generating their mature secreted forms. Caspase-1 can also induce cell death by pyroptosis^15^. Osteoblasts express inflammasome components, including the core protein of NLRP3 inflamasome, NALP3^16^. Activation of Caspase-1 leads to cell death by pyroptosis of osteoprogenitor cells^17^. Infection of osteoblastic cells with *Aa* induced production of IL-1β and IL-18 and apoptosis, both events mediated by the activation of NLRP3 inflammasome^18^. These events may affect bone turnover and inflammatory bone resorption *in vivo*. Cytokines processed by the effector caspase-1, particularly IL-1β, may modulate osteoclast differentiation and activity by direct effects on osteoclasts^19^ or by indirectly modulating the expression of RANKL (ligand for receptor activator of nuclear factor-kappa-B) by other cell types^20^.

There is only one study assessing the role of NLRP3 inflammasome in a *Pg*-colonization model of experimental periodontitis that reported a reduction in pro-inflammatory cytokine production and resorption of alveolar bone^21^; however we did not find any studies assessing the relevance of Caspase-1 in this disease model. This inceptive study intends to contribute to bridge this gap of knowledge, by providing insights into the role NLRP3 inflammasome and of Caspase-1 on inflammation and alveolar bone resorption in a murine model of bacterial-induced experimental periodontal disease.

## Materials and Methods

### Periodontal disease model

A total of 36 C57BL/6 male adult mice (age between 6 and 8 weeks) were used, including 12 wild-type (WT) mice, 12 mice genetically deficient (knockout) for Nalp3 (Nrlp3-KO), the central NLR protein in the NLRP3 inflammasome, and 12 mice genetically deficient for Caspase-1 (Casp1-KO). All mice were obtained from the Center for genetically modified and transgenic mice, School of Medicine at Ribeirao Preto-University of Sao Paulo (USP). Disruption of the targeted genes was verified at mRNA and protein level (Supplemental Fig. 1). This study was carried out in accordance with the principles stated by the Brazilian College of Animal Experimentation and was approved by the Ethical Committee on Animal Experimentation (protocol number 06/2014) of the School of Dentistry at Araraquara, UNESP-State University of Sao Paulo, Araraquara, SP, Brazil.

Experimental periodontal disease was induced in 18 mice, including 6 animals of each genotype (WT, Nlrp3-KO and Casp1-KO) by direct bilateral injections of a 3 uL PBS suspension of heat-killed *Aggregatibacter actinomycetemcomitans (Aa, JP1 serotype)* at 1×10^9^ UFC/mL^22,23^. 18 non-disease control mice (n = 6 for each genotype) received bilateral injections of the same volume of the PBS vehicle. These injections were performed under mild general anesthesia with isofluorane (Baxter Healthcare, Deerfield, IL) using a Hamilton-type microsyringe (33 gauge needle) three times/week for 4 weeks, directly in the gingival tissues at the palatal aspect between the first and second upper molars. All animals were euthanized by cervical dislocation 4 weeks after the first injection.

The maxillary bones were hemisected and submitted to microcomputed tomographic analysis of alveolar bone resorption. After scanning, the specimens were submitted to routine EDTA decalcification and processing to obtain paraffin-embeded tissue blocks for the histological and immunofluorescence analyses.

### In vitro studies

Primary M-csf-differentiated macrophages were derived from cells obtained from the marrow of long bones (femur and tibia) of WT, Nlrp3-KO and Casp1-KO mice as previously described^24^. These cells were plated in regular tissue culture-treated and calcium phosphate-coated (Osteologic, Corning-Costar, Corning, NY, USA) 96-well plates (1×10^4^ cells/well) and after 18 h, stimulated with 50 ng/mL of murine recombinant Rankl and 20 ng/mL of murine recombinant M-csf (Peprotech Inc, Rocky Hill, NJ, USA). Medium was changed and these stimuli re-applied at 72 h. Cultures were kept for an additional 48 h (a total of 5 days of osteoclastic differentiation). Cells grown in regular tissue culture-treated plastic were fixed with paraformaldehyde and permeabilized in saponin-containing buffer (BD Cytofix/Cytoperm, BD Biosciences, San Jose, CA, USA) and stained with AlexaFluor 488-conjugated phalloidin (Molecular Probes, ThermoFisher Scientific, Waltham, MA, USA) for 40 minutes, followed by DNA staining with DAPI (Sigma-Aldrich Co., St. Louis, MO, USA) for 5 minutes for the identification of actin ring formation. Total RNA was also isolated in parallel experiments for RT-qPCR. Both M-csf differentiated macrophages (20 ng/mL for 2 days) and Rankl/M-csf differentiated osteoclasts grown in regular 96-well tissue culture plates (1×10^5^ macrophages/well, 1×10^4^ bone marrow cells/well for osteoclasts) were lysed for RNA isolation. Macrophages were stimulated with 100 ng/mL of LPS (*E.coli* LPS, Sigma-Aldrich Co., St Louis, MO, USA) or with the same volume of PBS vehicle for 18 h. Cells grown on calcium phosphate-coated 96-well plates were lysed by incubation in 1% sodium hypochloride for 15 min. Three digital images from each well (covering > 80% of the well surface) of phalloidin/DAPI-stained and of calcium-phosphate coated plates were obtained at 40X magnification on an inverted digital fluorescence microscope (Evos fl, AMG Micro, ThermoFisher Scientific, Waltham, MA, USA). A trained examiner blind to the experimental conditions counted the number of osteoclasts (cells with evidence of actin ring formation and containing three or more nuclei) and measured the perimeter of the osteoclasts in the merged green/blue channel fluorescent images. In the images from calcium phosphate-coated wells, the area of exposed plastic was measured as indicative of resorbing activity. A trained examiner not aware of the experimental conditions performed these measurements using ImageJ software (v. 1.51 s, National Institutes of Health, USA – http://imagej.nih.gov/ij).

### Microcomputed tomography analysis (µCT scanning)

The hemimaxillae were initially fixed in 4% buffered formalin for 24 h and transferred to 70% alcohol until scanning using 56 kV, 300 mA and a 0.5 mm aluminum attenuation filter, with the resolution of the slices set to 18 µm using a µCT system (Skyscan, Aartselaar, Belgium). Tridimensional images were reconstructed and the resulting images were oriented in three planes (sagittal, coronal and frontal) in a standardized manner using anatomical landmarks with NRecon and DataViewer softwares (Skyscan, Aartselaar, Belgium). A standardized 5.4 mm^3^ region of interest (ROI) was set with 1.5×4.0×0.9 mm (vertical or cervico-apical x horizontal or mesio-distal x lateral or buccal-palatal). This cuboidal ROI was positioned on the central sagittal section (identified by the diameter of the root canal in the distal root of the first molar) using the following references: 1. cervical/coronal reference was the roof of the furcation area between mesial and distal roots of the upper first molar; 2. mesially we used the distal aspect of the mesial root of the first molar. The thickness of the ROI was set to 50 slices (900 µm) counted from this central section towards the palatal/medial direction on the sagittal plane. For the analysis, a standardized threshold of grey level was set to distinguish between non-mineralized and mineralized tissues. Considering that variations on the size and mineralization of the tooth structures included in the ROI were irrelevant among the different animals, the analysis assessed the percentage of mineralized tissue (MT) within the total volume (TV) of the ROI, presented as a ratio (MT/TV). A decrease on this ratio is interpreted as indicative of bone resorption.

### Histological analysis

The hemimaxillae with intact surrounding soft tissues were fixed in 4% buffered formalin for 48 h, decalcified in EDTA (0.5 M, pH 8.0) for 45 days at room temperature, and embedded in paraffin. Semi-serial sections of 4 µm thickness were obtained in the buccal-lingual (frontal plane) direction and stained with hematoxylin and eosin (H/E).

### Immunohistochemical staining of TRAP

Nine unstained semi-serial sections from each paraffin-embedded hemimaxillae spanning 1000 µm on the antero-posterior direction (sagittal plane, n = 6 animals/experimental condition and genotype) were used for detection of tartrate resistant acid phosphatase (TRAP) expression. Briefly, the sections were deparafinized in xylene and rehydrated in decreasing concentrations of ethanol. Endogenous peroxidase was blocked using 3% peroxide in methanol (5 min, RT), followed by antigen retrieval by heating (95–98 C) the sections in tris/EDTA buffer (pH 9.0) for 15 min and blocking (1 h, RT) of non-specific binding with 2% BSA. Primary antibody for TRAP (cat# ab191406, Abcam, Cambridge, MA, USA) was diluted (1:100) in background-reducing solution (Dako-Agilent, Santa Clara, CA, USA) and incubated overnight at 4 C. The detection reaction was developed using a HRP-DAB visualization system (LSAB2, Dako-Agilent, Santa Clara, CA, USA). Osteoclasts were identified as large TRAP-positive cells, containing three or more nuclei located in the vicinity of the alveolar bone. A single trained examiner who was blind to the coding identifying the experimental groups and genotypes counted the osteoclasts (at 40X magnification) located from the apical portion of the palatal root of the first molar along the periodontal ligament upwards to the alveolar bone crest and towards the center of the palate, adjacent to the depression on the palatal bone associated with the major palatine artery and nerve.

### Immunofluorescence analysis of the inflammatory infiltrate

Unstained semi-serial sections of 4 µm thickness (9 sections per animal and experimental group, spanning 900 µm in the sagittal plane) were deparaffinized in two changes of xylenes for 15 and 5 minutes, and then dehydrated in 100% ethanol for 2 minutes. The slides were then rehydrated through a graded ethanol series (95% and 70%) for 2 minutes each, followed by a wash in distilled water for 1 minute, and then placed in Trilogy buffer (Cell Marque, Hot Springs, AR, USA) at 96.5 C for 25 minutes. Slides were cooled in a distilled water bath for 5 minutes, and rinsed in 1x TBS (Tris-Buffered Saline). Sections were permeabilized with 0.1% Triton X-100 (ThermoFisher Scientific, Waltham, MA, USA) and blocked in 1X PBS-T (0.5% Tween 20) containing 10% normal goat serum (Life Technologies, ThermoFisher Scientific, Waltham, MA, USA) at room temperature for 30 minutes. Slides were washed with 1x TBS and incubated with primary antibodies diluted in 1x TBS for 24 h at 4 C. Primary antibodies and dilutions used were as follows: CD45 at 1:200 (rat IgG, purified anti-mouse CD45, Biolegend, San Diego, CA, USA), for ‘general’ leukocyte infiltration, and Ly6G at 1:100 (rat IgG, purified anti-mouse Ly6G, Biolegend, San Diego, CA, USA), for specific neutrophil (PMN) staining. Negative control included irrelevant rat IgG at 1:100 dilution. Tissues were washed three times for 5 minutes each in 1x TBS and incubated with goat anti-rat secondary antibody conjugated with AlexaFluor 594 (ThermoFisher Scientific, Waltham, MA, USA) at 1:650 and 1:200, respectively, for 2 hours, at room temperature. The DNA dye 4’,6-diamidino-2-phenylindole (DAPI) was used to counterstain cell nuclei. Images were obtained on an EVOS fl digital inverted microscope (ThermoFisher Scientific, Waltham, MA, USA), and the median fluorescence intensity (MFI) in the red channel was determined using Image J software (v. 1.51 s, National Institutes of Health, USA – http://imagej.nih.gov/ij).

### Quantitative Reverse-Transcription Real-Time PCR

Total RNA was extracted from bone marrow-differentiated macrophages and Rankl/M-csf-differentiated osteoclasts (n = 7 samples from independent experiments, assessed in triplicate) using affinity columns (RNAqueous-4PCR, Ambion Inc, Invitrogen Corp., Foster City, CA, US) according to the manufacturer’s protocol. The quantity and purity of total RNA were determined by UV spectrophotometry and by the 260/280 nm ratio, respectively. 500 ng of total RNA was converted into cDNA using random hexamer primers and moloney leukemia virus reverse transcriptase in a reaction volume of 20 uL (High Capacity cDNA Synthesis kit, Applied Biosystems, Invitrogen Corp., Foster City, CA, USA). The qPCR reactions were performed in a 20 µL total volume reaction, including TaqMan qPCR mastermix (TaqMan Fast Advanced, Applied Biosystems, Invitrogen Corp., Foster City, CA, USA), cDNA template, deionized water, and mouse-specific pre-designed and optimized sets of primers and probe (TaqMan gene expression assays, Applied Biosystems, Invitrogen Corp., Foster City, CA, USA; supplemental table 1). Cycling conditions were pre-optimized by the supplier of the sets of primers/probe and master mix, and 40 cycles were run on a StepOne Plus qPCR thermocycler (Applied Biosystems, Invitrogen Corp., Foster City, CA, USA). Relative levels of gene expression were determined by the ∆(∆Ct) method using the thermocycler’s software and automated detection of the Ct. Expression of 18 S RNA in the same samples was used to normalize the results of the target genes.

### Statistical analysis

The statistical analysis was performed using Prism 8.3 (GraphPad Software LLC, San Diego, CA, USA). Central tendency and dispersion measures were calculated from different experiments. Comparisons between the experimental conditions (control/vehicle *versus* disease/stimulated) within each genotype background (WT, Nlrp3-KO, Casp1-KO) were performed using unpaired t-tests with Welch’s correction. Comparisons among the different genotypes (WT, Nlrp3-KO, Casp1-KO) in each experimental condition (control/vehicle *versus* disease/stimulated) were performed using Brown-Forsythe and Welch’s ANOVA followed by post-hoc test for pairwise comparisons. Significance level was set at 95% (p < 0.05) for all analyses.

## Results

### Inflammatory bone resorption is attenuated in Casp1-KO, but not in NLRP3-KO mice

**Figure 1:**
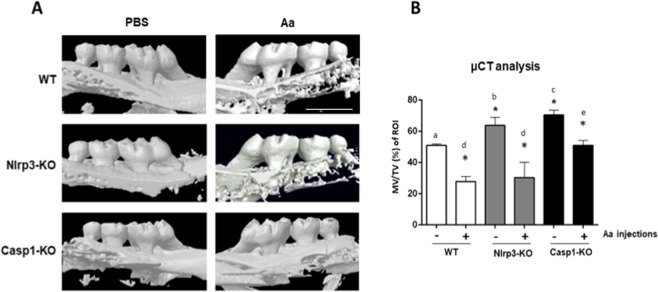
(**A**) Representative images of tridimensional reconstructions of microcomputed tomography scans of the hemimaxillae from WT, Nlrp3-KO and Casp1-KO mice, according to the experimental condition (non-disease control/PBS-injected or diseased/Aa-injected). Scale bar of 1 mm. (**B**) Alveolar bone resorption was evaluated as the change in the ratio between mineralized tissue (MT) to the total volume (TV) of the standardized region of interest (ROI). Images from 6 different animals (12 hemimaxillae, n = 6) from each genotype and experimental condition were analyzed. Bars represent means and vertical lines standard deviations. The asterisk (*) indicates a significant difference between control (PBS-injected) and diseased (Aa-injected) specimens in each genotype (WT, Nlrp3-KO and Casp1-KO) (unpaired t-test with Welch’s correction). Different letters indicate statistically significant differences among control (PBS-injected, a-c) or diseased (Aa-injected, d-e) specimens across all three genotypes (Brown-Forsythe and Welch’s ANOVA followed by Dunnett’s multiple comparisons test).

Interestingly, µCT analysis showed a greater volume of mineralized tissue (MT) in non-disease control (PBS-injected) Nlrp3-KO and Casp1-KO animals in comparison with WT mice. Injection of heat-killed Aa effectively induced alveolar bone resorption in all genotypes, but the severity of resorption was significantly attenuated in Casp1-KO mice (Fig. 1A,B). These results suggest that Nlrp3 and Caspase-1 may have a role in physiological bone turnover and that Caspase-1, but not Nlrp3, has a role in promoting inflammatory bone resorption.

### Osteoclast number is increased in Casp-1 KO mice

**Figure 2:**
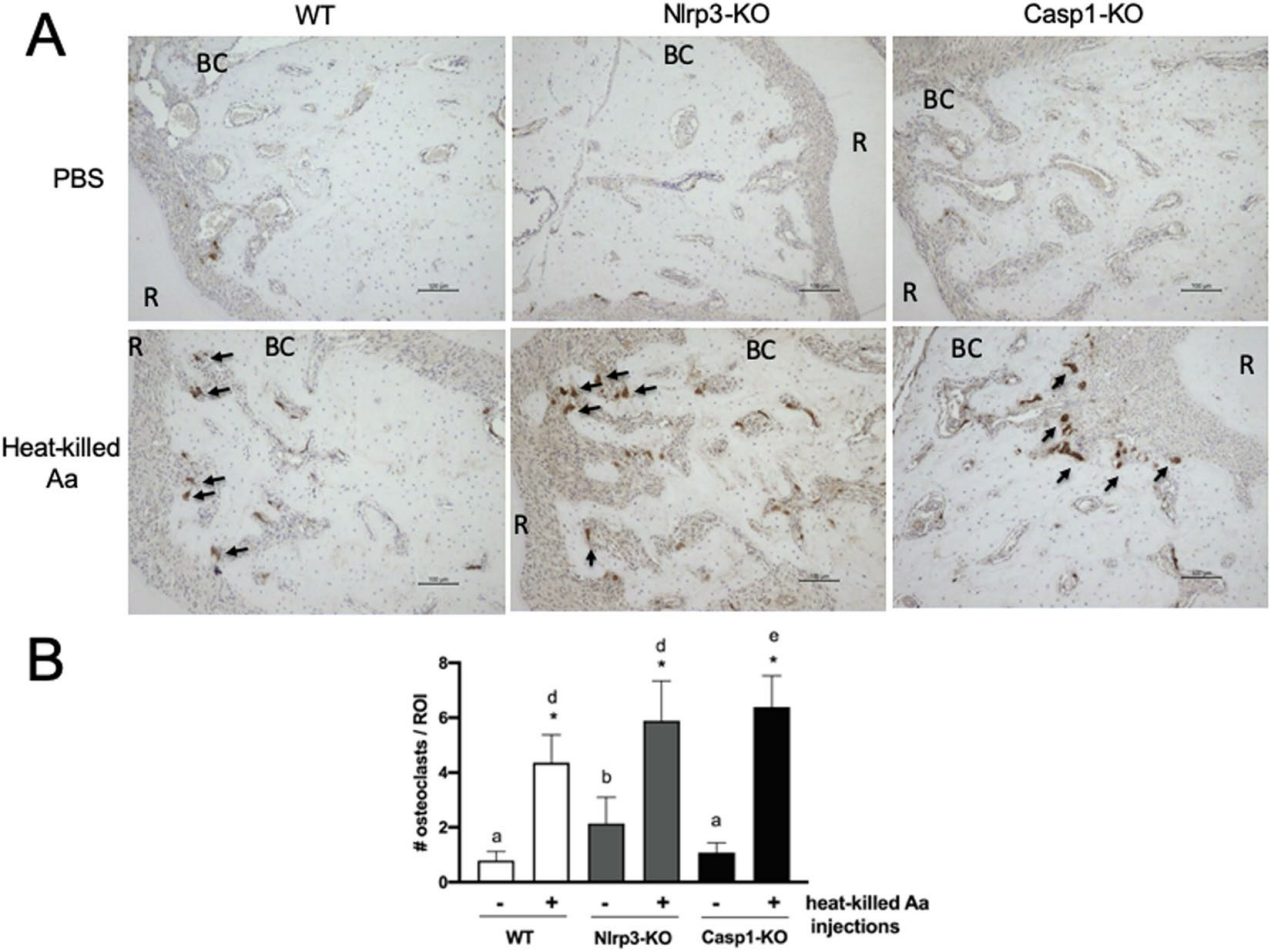
(**A**) Representative images of immunohistochemistry for TRAP in the periodontal tissues, both in non-disease control (PBS-injected) and diseased (Aa-injected) mice, according to the different genotypes (WT, Nlrp3-KO and Casp1-KO). Large, multinucleated positively cells near the alveolar bone were counted as osteoclasts (40×, arrows indicate osteoclasts, BC the bone crest, and R the palatal root of the first molar). (**B**) Bars indicate means and vertical lines the standard deviation of the number of osteoclasts according to the experimental condition (non-disease control / PBS-injected and diseased/Aa-injected) and genotype (WT, Nlrp3-KO and Casp1-KO). The quantification was performed in 9 different and non-sequential sections spanning 1000 µm on the sagittal plane (antero-posterior) from 6 different animals in each experimental condition (non-disease control/PBS-injected and diseased/Aa-injected) and genotype (WT, Nlrp3-KO and Casp1-KO). The asterisk (*) indicates a significant difference between control (PBS-injected) and diseased (Aa-injected) specimens in each genotype (WT, Nlrp3-KO and Casp1-KO) (unpaired t-test with Welch’s correction). Different letters indicate statistically significant differences among control (PBS-injected, a-b) or diseased (Aa-injected, d-e) specimens across all three genotypes (Brown-Forsythe and Welch’s ANOVA followed by Dunnett’s multiple comparisons test).

Considering the attenuation of bone resorption in Casp1-KO mice, the increase in the number of osteoclasts associated with the induction of experimental periodontal disease was surprisingly similar in all genotypes. Osteoclasts are identified as large, positively-stained multinucleated cells located in the vicinity of alveolar bone (indicated by arrows in Fig. 2A). These results indicate that osteoclast differentiation in this inflammatory microenvironment is not affected by the lack of Nlrp3 or Caspase-1. In fact, counter-intuitively lack of Caspase-1 significantly increased osteoclast numbers, but not bone resorption (Fig. 2B). There is a slight increase in osteoclast numbers in Nlrp3-KO mice, but it was not statistically significant.

### Infiltration of PMNs is attenuated in Casp1-KO mice

**Figure 3:**
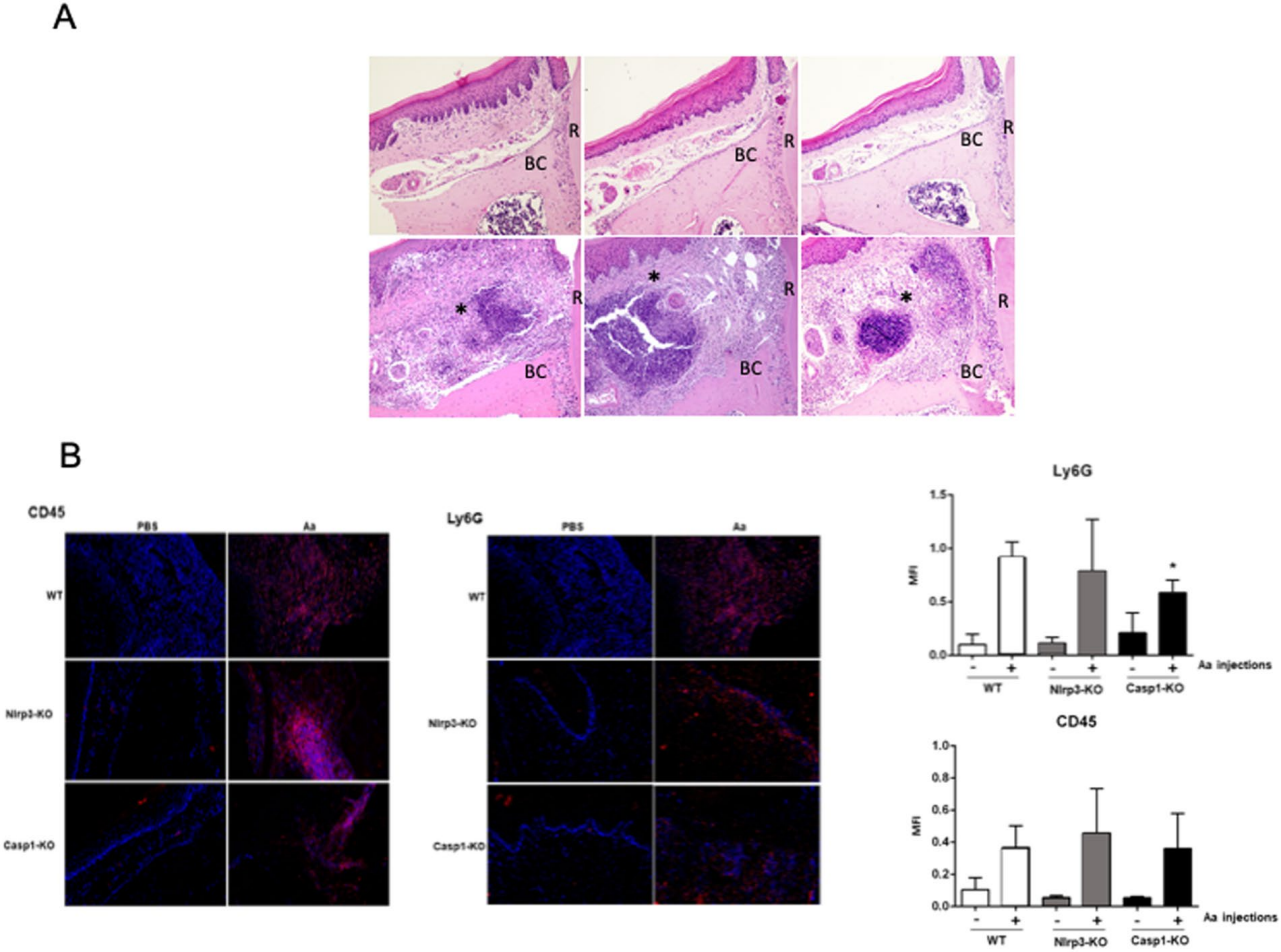
(**A**) Representative images of H/E-stained sections of each experimental group (non-disease control/PBS-injected or diseased/Aa-injected) according to the genotype (WT, Nlrp3-KO or Casp1-KO) at 100X magnification (BC, bone crest, R, palatal root of the first molar, * indicates inflammation in the injection area) (**B**) Representative images of immunofluorescence detection of the pan-leukocyte marker CD45 and the neutrophil marker Ly6G in the gingival tissues of WT, Nlrp3 and Casp1-KO mice, according to the experimental condition (non-disease control/PBS-injected or diseased/Aa-injected). Nuclei were counterstained with DAPI. The results for the quantitation of mean fluorescence intensity (MFI) in the red channel (AlexaFluor 594) of CD45 or Ly6G according to the experimental condition and genotype are presented in the graphs. Nine semi-serial sections from each animal and experimental condition spanning 900 µm in the sagittal (antero-posterior) plane. Bars represent means and vertical lines the standard deviation of MFI values from at least 4 animals per group and experimental condition (Brown-Forsythe and Welch’s ANOVA followed by Dunnett’s multiple comparisons test).

Induction of experimental periodontal disease was associated with a marked increase in inflammatory cell infiltrate in WT, Nlrp3-KO mice and Casp1-KO mice (Fig. 3A, inflamed area corresponding to the site of injections indicated by an asterisk, ‘BC’ indicates the alveolar bone crest and ‘R’ indicates the palatal root of the upper first molar, H/E stained images). Immunofluorescence analysis (Fig. 3B) shows a marked increase on leukocyte (CD45 + ) infiltration in the gingival tissues of WT, Nlrp3-KO and Casp1-KO mice. There was no statistically significant difference on the overall inflammatory infiltrate (CD45 + cells) in diseased gingival tissues among the three genotypes; however there was a significant decrease on the PMN (Ly6G + ) infiltrate in Casp1-KO mice, which also had the least relative increase of PMN infiltration with the induction of experimental periodontal disease.

### ***LPS-induced expression of Il-10, Il-12 and Tnf-α*** is reduced in Nlrp3- and Casp1-deficient macrophages

**Figure 4:**
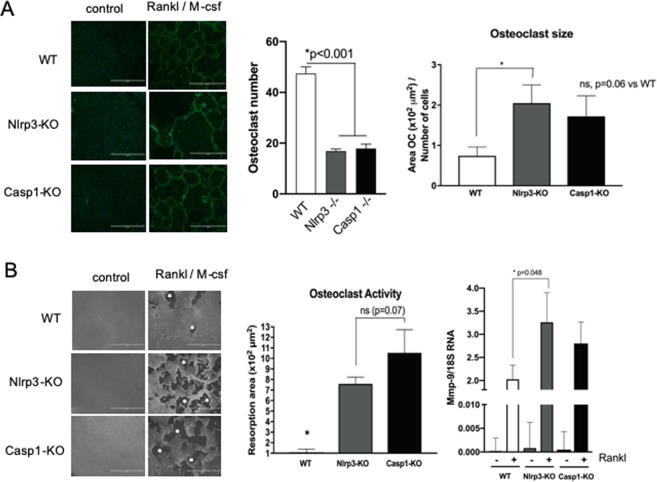
Expression (mRNA) of selected candidate genes associated with macrophage phenotype and inflammation. Bone marrow-derived macrophages from WT, Nlrp3- and Casp1-KO mice were differentiated with M-csf (20 ng/mL) over 48 h and then stimulated with LPS (100 ng/mL) for 4 h. Unstimulated cells were treated with the same volume of PBS vehicle. Expression of Il-10, Il-12 and Tnf-α was determined by RT-qPCR using Taqman chemistry and pre-designed sets of primers and probe. Bars represent means and vertical lines the standard deviation of normalized target gene expression (n = 7 independent experiments, assessed in triplicate). The asterisk (*) indicates a significant difference in comparison with unstimulated cells (Unpaired t-test with Welch’s correction). Double asterisks (**) in the bracketed columns indicate significant difference (Brown-Forsythe and Welch’s ANOVA followed by Dunnett’s multiple comparisons test).

Considering that bacterial LPS is a major disease-inducing antigen in periodontal diseases, we assessed the role of Nlrp3 and Caspase-1 on the expression of the inflammation- and macrophage phenotype-associated genes Il-10, Il-12 and Tnf-α in primary bone marrow-derived macrophages. In WT and Nlrp3-deficient macrophages, LPS stimulation caused a statistically significant increase in Il-10, Il-12 and Tnf-α, whereas in Casp1-deficient macrophages only Il-10 was significantly induced. In comparison with WT macrophages, expression of all three candidate genes was markedly reduced in Nlrp3- and Casp1-deficient macrophages, although the inhibition of Il-12 expression was not statistically significant (Fig. 4).

### Caspase-1 and Nlrp3-deficiency increases the activity of Rankl-derived osteoclasts in vitro

**Figure 5:**
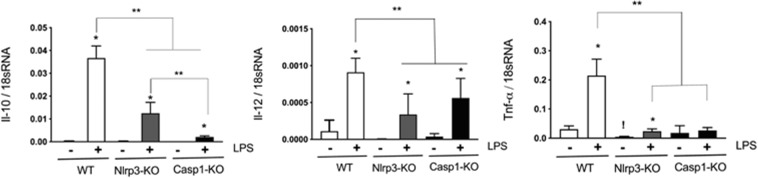
(**A**) Representative images of actin ring formation in osteoclasts differentiated from bone marrow-derived macrophages isolated from WT, Nlrp3-KO and Casp1-KO mice (40×, scale bar 1000 µm). Bars represent means and vertical lines the standard deviation of osteoclast numbers and of the perimeter normalized to the number of osteoclasts assessed. Asterisk (*) indicates a significant difference between bracketed bars (Brown-Forsythe and Welch’s ANOVA followed by Dunnett’s multiple comparisons test). (**B**) Representative images of pit assay using Calcium phosphate-coated plates indicating the resorptive activity (clear area of exposed plastic substrate indicated by asterisks results from the removal of Calcium phosphate coating by the osteoclasts) of osteoclasts according to the genotype of the mice (40×, scale bar 1000 µm). Quantification of resorptive activity and Mmp-9 mRNA expression are depicted in the graphs. Bars represent means and vertical lines the standard deviation (n = 7 independent experiments, analyzed in triplicate) of the area of exposed plastic substrate (top) and normalized Mmp-9 mRNA expression (bottom). The asterisk (*) indicates a significant difference between resorbed area in WT osteoclasts in comparison to osteoclasts derived from Nlrp3- and Casp1-KO mice (Brown-Forsythe and Welch’s ANOVA followed by Dunnett’s multiple comparisons test).

Macrophages from the bone marrow of WT, Casp1-KO and Nlrp3-KO mice differentiated into osteoclasts when treated with Rankl and M-csf over 5 days. The osteoclasts derived from Nlrp3-KO mice were significantly larger, which justifies the reduction in number of osteoclasts due to the limited cell growth area (Fig. 5A). Moreover, osteoclasts differentiated from the bone marrow cells of both Nlrp3- and Casp1-KO mice had a significantly greater resorbing activity than the osteoclasts derived from the bone marrow of WT mice (Fig. 5B, the clear areas indicated by asterisks in the images result from resorption of the calcium phosphate coating by the osteoclasts). Mmp-9 expression was also increased upon osteoclast differentiation by treatment with RANKL over 5 days. Osteoclasts derived from the bone marrow of Nlrp3-KO mice had significantly greater expression of Mmp-9 in comparison with osteoclasts derived from the bone marrow of WT animals (Fig. 5B).

## Discussion

To our knowledge, this is the first study to assess the role of Caspase-1 in an *in vivo* model of bacterial-induced periodontal disease. The results indicate that the Nlrp3 inflammasome does not have a relevant role in the inflammatory bone resorption in this model. These results should be considered in the context of an inceptive study, generating insights and questions that shall be further explored in future studies. Inactivation of Caspase-1 gene significantly attenuated inflammatory bone resorption; however this effect was not accompanied by a reduction in the number of osteoclasts, on the inflammatory infiltrate or on the transcription of selected candidate genes associated with inflammation and mineralized and non-mineralized soft tissue degradation. These results are in contrast with those of a study that used a *Porphyromonas gingivalis* oral colonization model of experimental periodontitis, which showed a significant attenuation of bone resorption in Nlrp3-deficient mice^21^. The differences in experimental design (oral colonization with live bacteria *versus* direct injection of heat killed bacteria), in the bacterial species used (*P.gingivalis versus A.actinomycetemcomitans*) and in the methods of assessment of bone resorption (histomorphometric linear measurements X µCT tridimensional volumetric analysis) may account for the discrepancy. Importantly, the limitations of the experimental approach in the present study have to be considered when interpreting the data, particularly the lack of data on protein production (most notably of IL-1β, the prototypical NLRP3 inflammasome-activated cytokine) and of data exploring the biological mechanisms involved in the observed phenotype.

Interestingly, most *in vivo* and *in vitro* studies related with periodontal disease used the Gram-negative bacterial species *Porphyromonas gingivalis* (*Pg*) as the exogenous stimulus. In spite of its relevance to periodontitis, *Pg* is not the only bacterial species associated with periodontal diseases in the subgingival dental biofilm; and some studies have demonstrated that other microbial species, such as *Streptococcus sanguis*^25^, *Mycoplasma salivarium*^26^, *Fusobacterium nucleatum* (*Fn*)^27^ and *Aggregatibacter actinomycetemcomitans* (*Aa*)^28^ also regulate the expression of NLRP3 inflammasome components and of the inflammasome-processed cytokines. In fact, there is evidence that NLRP3 is differentially regulated by secreted products from supra and subgingival biofilms^29^, as well as conflicting information regarding the inhibitory^30–33^ or stimulatory^33–38^ effect of *Pg* and *Pg*-derived antigens on NLRP3 expression and activation. These conflicting reports are related with the assessment of different cell types, *Pg*-derived antigen used, experimental design (e.g., second signal for inflammasome activation, experimental period, outcomes assessed) and on the presence or absence of hypoxia.

In this study we used heat-killed Gram-negative bacteria associated with periodontal disease in humans to avoid issues with possible fluctuation of bacterial cell viability and also issues with adherence/colonization in the oral environment. *In vitro* studies show that *Aggregatibacter actinomycetemcomitans* (Aa, used in this study) induces expression of NLRP3 and IL-1β by human monocytes^39^ and human PBMCs^40^. Some studies report that leukotoxin secreted by Aa is a crucial virulence factor mediating the induction of IL-1β and IL-18^41^; however, another *in vitro* study using human monocytes infected with mutant strains of Aa (knockout for leukotoxin and cytolethal dystending toxin genes) also showed increased expression of IL-1β, IL-18 and NLRP3, suggesting that other molecules derived from Aa may activate inflammasomes^40^. This supports the possibility of inflammasome activation in our model using heat-killed Aa, which is further indicated by our *in vitro* data (Supplemental Fig. 2) demonstrating the agonistic effect of heat-killed Aa on bone marrow-derived macrophages.

The goal of our model was to induce an inflammatory response and the associated inflammatory alveolar bone resorption, the two major hallmarks of periodontal disease. This model provides the two signals that are required for activation of the Nlrp3 inflammasome: exogenous microbial-derived PAMPs, which triggers the production of cytokine precursors (e.g., pro-IL1); and a second signal, represented by the interaction of DAMPs with their PRRs (e.g., RAGE, HMGB1). The two-signal model of activation of NLRP3 was already demonstrated in the context of periodontal disease by stimulating primary gingival epithelial cells with *Porphyromonas gingivalis*, which resulted in downregulation of NLRP3 expression and increase of pro-interleukin-1β expression. Increased secretion of interleukin-1β was only detected upon stimulation with extracellular ATP as the danger signal/second signal^34^.

In addition to the presence of specific ligands/activators of NLRP3, the chronic inflammatory microenvironment of periodontal disease is characterized by high levels of reactive oxygen species (ROS), hypoxia^33^ and also by tissue degradation, with accumulating DAMPs. Both ROS and DAMPs (which may also induce production of ROS) can activate multiple inflammasomes besides NLRP3, including AIM2, NLRP1, NLRC4 and NLRP6^42^. Thus, inflammasome activation may derive from direct recognition/interaction of bacterial and host-derived ligands by the inflammasome central/sensor proteins coupled with the detection of cell changes induced by external microbial/stress stimuli^43^. Specifically regarding periodontal disease, there is scarce information available. Increased gene expression of NLRP3 and NLRP2, but not of ASC-1, was reported in the presence of periodontal disease in humans, and these increased levels of inflammasome core genes were correlated with increased mRNA of IL-1β and IL-18, cytokines processed by the inflammasomes^12^. Increased expression of NLRP3 and AIM2 in the gingival tissues of patients with periodontal disease is positively correlated with the levels of IL-1β and IL-18, suggesting that various inflammasomes may participate in the microbial-induced inflammation in periodontal diseases^35^. In our experimental model, it is important to consider that only the NLRP3 inflammasome was disrupted in Nlrp3-KO mice (Supplemental Fig. 1) and this may cause a compensatory activation of the other functional inflammasomes (e.g., NLRC4, AIM2), with a shift in the biological effects. In fact, induction of experimental arthritis in Il10-KO mice caused a significant increase in the expression of Nalp3, Aim2 and Caspase-1^44^. Nevertheless, the lack of functional Nlrp3 did not affect the inflammatory infiltrate and alveolar bone resorption in this model. In contrast, genetically modified mice with a global gain of function mutation of Nlrp3^45^ demonstrated increased production of proinflammatory mediators, which was associated with altered bone turnover and reduced bone mass; however when the Nlrp3 gain of function mutation was limited to osteoclasts there was no increase in proinflammatory cytokines accompanying the decrease of bone mass^46^. Rankl-induced osteoclast differentiation *in vitro* was not affected in Caspase-1 and Nlrp3-deficient macrophages, but cell size, resorbing activity and Mmp-9 expression of the resulting osteoclasts were all significantly increased in comparison with bone marrow-derived macrophages from WT mice.

LPS induction of Il-10, Il-12 and Tnf-α was significantly reduced in Nlrp3- and Casp1-deficient macrophages, suggesting that Nlrp3 influences Tlr4-associated gene expression. This is surprising, considering that a major biological function of inflammasomes is the posttranslational processing of cytokines and that LPS stimulation is usually considered as a first signal priming macrophages for inflammasome activation. In our experiments, the 18h-stimulation period may have allowed for the production of molecules that provide an autocrine/paracrine second stimulatory signal for the activation of inflammasomes. Prolonged stimulation of macrophages with LPS has been recently shown to promote the maturation and secretion of IL-1β independently of Nlrp3 activation^47^. However, both constitutive and LPS-induced expression of Il-10 is reported to be significantly reduced in Nlrp3-deficient murine macrophages, even with a shorter period of stimulation (4 h)^48^. The mechanism associated with this possible crosstalk between TLR signaling and the NLRP3 inflammasome is unknown, but at least for IL-10 it is independent of the major signaling pathways activated downstream of TLR4 (p38 and ERK MAPKinases and NF-kB)^48^. It is also possible that lack of LPS-induced posttranslational modifications of Nlrp3^49–51^ in the macrophages derived from Nlrp3-KO mice is involved in inhibition of cytokine expression. Moreover, we cannot rule out the involvement of non-canonical Caspase-11 inflammasome in regulating the responses of macrophages to LPS in the cytosol^52,53^, although it is still unclear how LPS may enter the cells. Interestingly, the Caspase-11 non-canonical inflammasome can also induce a non-canonical activation of the Nlrp3 inflammasome^54^.

Various possible scenarios may be implicated in these intriguing and apparently contradictory findings *in vitro*. *In vivo*, osteoclast-precursor cells may be derived from a different population of precursor cells influenced by a much more complex microenvironment that includes other inflammatory and stromal cell types, as well as multiple biologically-active molecules, including cytokines, chemokines, lipid-derived mediators, as opposed to the more simple and defined microenvironment of the *in vitro* experiments. Also, the experimental periods *in vitro* are not commensurable with the experimental periods of the *in vivo* experiment. These possibilities will be further investigated in subsequent studies.

Since Caspase-1 is the main downstream effector of all inflammasomes, Casp1-KO mice may be considered as representative of a global inflammasome loss of function, which in our experimental model was associated with a significant attenuation of inflammatory bone resorption, indicating that Caspase-1 activation in the microenvironment of periodontal disease has a relevant role in the inflammatory bone resorption. We speculate that activation of other Caspase-1 activating inflammasomes may compensate the inactivation of Nlrp3 in Nlrp3-KO mice, which may account for the lack of influence on bone resorption associated with experimental periodontitis in these mice. Strikingly, inflammatory infiltrate, osteoclastogenesis and expression of candidate inflammatory genes were not affected by the lack of Caspase-1 in our experimental model, which contrasts with the phenotype of attenuated bone resorption. Speculatively, lack of Caspase-1 may cause a functional impairment and/or a phenotypical change through both direct and indirect influences on the cascade of events in inflammatory cells and osteoclasts; such as a shift towards the activation of the non-canonical Caspase-11 inflammasome by bacteria phagocytosed by macrophages in our experimental model. The significant impairment in PMN infiltration observed in Casp1-KO mice suggests that PMNs may play a role in alveolar bone resorption in this model and supports a phenotypical change in the inflammatory response. Interestingly, our *in vitro* data indicates that RANKL-induced osteoclastic differentiation is not affected in Casp1-deficient macrophages, which supports the *in vivo* finding of a similar number of TRAP-positive osteoclasts in WT and Casp1-KO mice. However, *in vitro* RANKL-differentiated osteoclasts derived from bone marrow macrophages from Nlrp3- and Casp1-deficient macrophages were larger and presented significantly increased resorptive activity, which would be expected to increase bone resorption *in vivo*. It is possible that this discrepancy is due to the presence of other biologically active mediators (besides Rankl and M-csf used *in vitro*) and cell types in the microenvironment *in vivo*, which may have reduced the resorbing activity. We speculate that osteoclast differentiation and activation are distinct processes that may be regulated independently. These possibilities will be explored in future experiments. In addition, these results indicate that other inflammatory pathways are involved in the pathogenesis of periodontal diseases, as inflammation and alveolar bone resorption were not completely abrogated in either Nlrp3- or Casp1-KO mice.

The suggestion is that inflammasome activation may affect bone turnover indirectly (i.e., via modulation of the level of active proinflammatory mediators) or directly (i.e., via an osteoclast-specific effect). The osteoclast-specific effect may not be necessarily associated with change in osteoclast number (i.e., osteoclast differentiation), but rather with increased osteoclast activity, as indicated by the *in vitro* experiments (Fig. 5A,B). On the other hand, selective blockage of Nlrp3 *in vitro* has been shown to reduce RANKL-induced osteoclastogenesis *in vitro*^44^. These contrasting results may be associated with differences in the experimental approach, particularly the use of bone marrow-derived macrophages from Il10-KO mice and the criterium used to identify the osteoclasts among the TRAP-positive cells, as cell size and the presence of three or more nuclei were not considered. Of note, we did not assess the infiltration of macrophages, important osteoclast precursors and prototypical inflammasome-expressing cells, in the gingival tissues. It is possible that changes in macrophage infiltrate and/or phenotype play a role in the observed phenotype of Nlrp3- and Casp1-KO mice. The influence of Nlrp3 inflammasome and Caspase-1 activities on *in vitro* chemotaxis and infiltration of macrophages *in vivo*, as well as on the phenotype of macrophages need to be explored in subsequent studies.

In summary, NLRP3 inflammasome did not play a significant role in inflammation and bone resorption in the heat-killed Aa-induced periodontal disease model; whereas lack of Caspase-1 attenuated inflammatory bone resorption and the infiltration of PMNs. Taken in the context of an inceptive study, the apparent contradictions and insights from the data presented are stimulating of further research into biological mechanisms by which inflammasomes can influence destruction of both soft and mineralized connective tissues in chronic inflammatory conditions associated with host-microbial interactions.

## Supplementary information

Supplementary dataset 1.

## Data Availability

The datasets generated during and/or analyzed during the current study are available from the corresponding author on reasonable request.
