# Supplementary material for: Relevance of Caspase-1 and Nlrp3 Inflammasome on Inflammatory Bone Resorption in A Murine Model of Periodontitis
Source: Sci Rep. 2020 May 8;10:7823. doi: 10.1038/s41598-020-64685-y (PMC7210885; doi:10.1038/s41598-020-64685-y)
Supplement: Supplementary file 1 — Supplementary dataset 1. [file 41598_2020_64685_MOESM1_ESM.docx]

**Supplemental data**

**RELEVANCE OF CASPASE-1 AND NLRP3 INFLAMMASOME ON INFLAMMATORY BONE RESORPTION IN A MURINE MODEL OF PERIODONTITIS**

Fernanda R. G. Rocha

,Andrea E. Delitto

Joao A Chaves de Souza

Laura A. González-Maldonado

Shannon M. Wallet

Carlos Rossa Jr

**Supplemental Table 1** – TaqMan Gene Expression Assays used in the assessment of gene expression by RT-qPCR

| **Gene symbol** | **RefSeq / GenBank mRNA** | **Exon boundary** | **Assay location (bp)** | **Amplicon length**  **(bp)** | **Assay ID** |
| --- | --- | --- | --- | --- | --- |
| Il10 | NM_010548.2 | 4 - 5 | 515 | 136 | Mm01288386_m1 |
| Il12a | AF128210.1 | 5 – 6 | 595 | 58 | Mm00434169_m1 |
| Tnf | NM_013693.3 | 1 – 2 | 352 | 81 | Mm00443258_m1 |
| Nlrp3 | NM_145827.3 | 2 – 3 | 499 | 84 | Mm00840904_m1 |
| Casp1 | NM_009807.2 | 3 – 4 | 532 | 99 | Mm00438023_m1 |
| Rn18s | NR_003278.3 | N/A | 217 | 115 | Mm04277571_s1 |

**
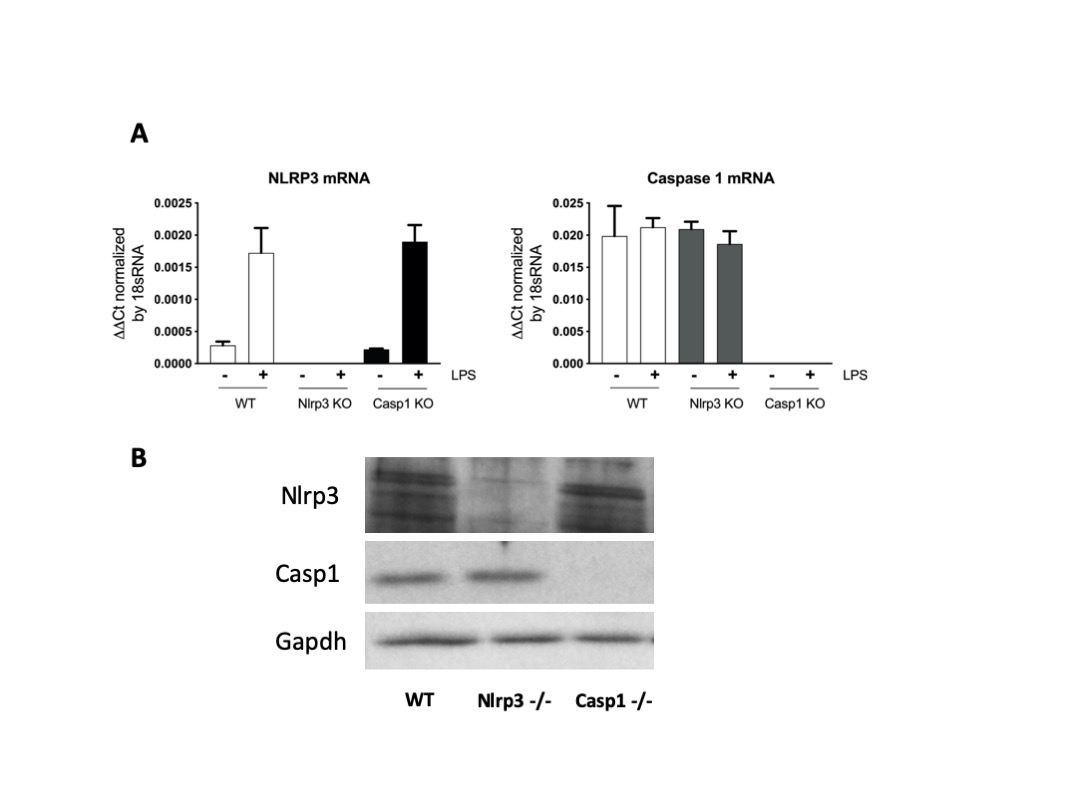
**

**Supplemental figure 1– Expression of Nlrp3 and Casp1 in bone marrow cells from WT, Nlrp3-KO and Casp1-KO mice.** **(A)** Bone marrow cells were cultured in DMEM supplemented with antibiotics and heat-inactivated FBS (10% vol:vol) for 3 days and then stimulated with 100 ng/mL of *E.coli* LPS (or the same volume of PBS vehicle) for 18 h. Total RNA was purified from cell lysates, and 500 ng were used for cDNA synthesis using random hexamers as primers. RT-qPCR reactions were performed using TaqMan chemistry and TaqMan Gene Expression Assays for Nlrp3, Caspase-1 and 18S RNA. Bars represent means and vertical lines standard deviations from cultures of bone marrow cells from 5 different mice in each genotype, assessed in triplicate. **(B)** Expression of Nlrp3 and Caspase 1 in bone marrow cells derived from WT, Nlrp3-KO and Casp1-KO mice. Bone marrow cells were cultured in DMEM supplemented with antibiotics and heat-inactivated FBS (10% vol:vol) for 3 days. Cells were lysed in RIPA buffer supplemented with proteinase and phosphatase inhibitors and 25 µg of proteins were separated by SDS-PAGE. Proteins were transferred onto nitrocellulose membranes, which were incubated with primary antibodies for Nlrp3, Caspase-1 and Gapdh. Image is representative of two independent experiments, performed using cells from 2 different mice of each genotype.

**
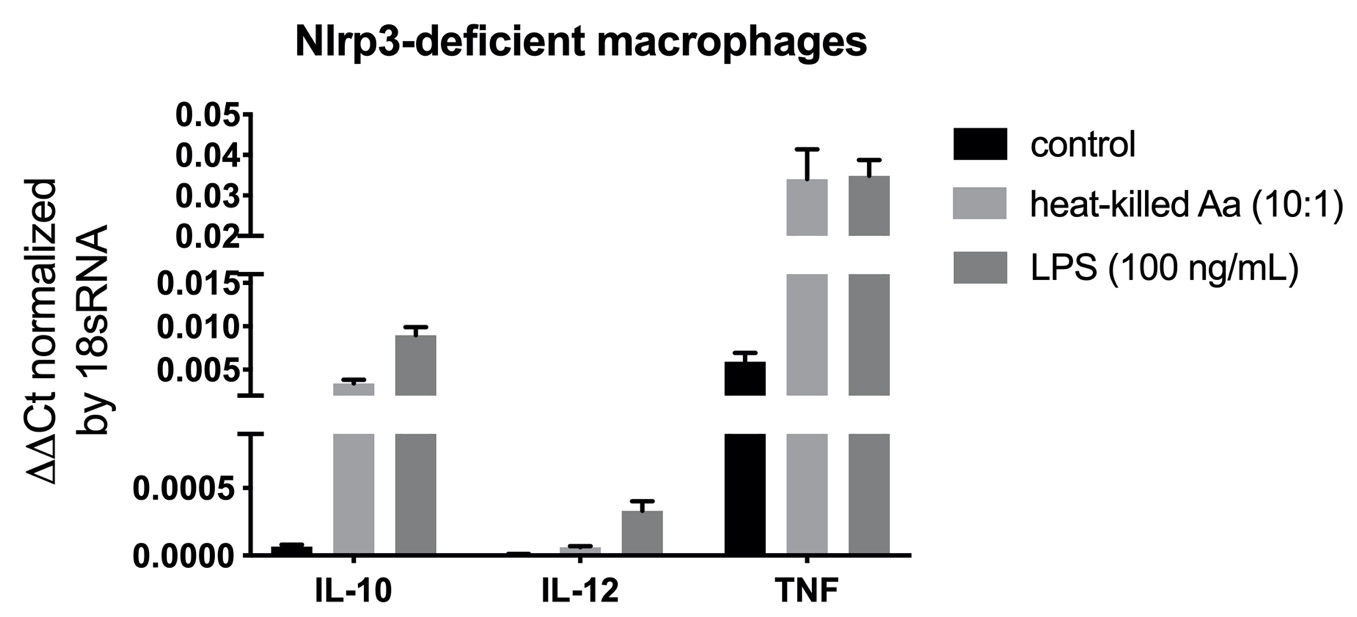
**

**Supplemental figure 2– Expression of selected target genes in Nlrp3-deficient macrophages after stimulation with *E.coli* purified LPS and heat-killed Aa**. Bone marrow derived cells were differentiated into macrophages with M-csf (20 ng/mL) over 2 days. After de-induction in low FBS-containing medium for 8 h, cells were stimulated with *E.coli* LPS (100 ng/mL) or heat-killed Aa (ratio 10 bacteria / macrophage) for 4 h. Control cells were treated with the same volume of PBS vehicle. Gene expression was assessed by RT-qPCR using pre-designed TaqMan Gene Expression Assays and normalized for the expression of 18S RNA. Data from 4 independent experiments, analyzed in triplicate.
